# Palliative care professionals’ willingness to perform euthanasia or physician assisted suicide

**DOI:** 10.1186/s12904-015-0058-3

**Published:** 2015-11-14

**Authors:** Julia Zenz, Michael Tryba, Michael Zenz

**Affiliations:** Ruhr-University Bochum, Universitätstr. 150, D 44801 Bochum, Germany; Clinic for Anaesthesiology, Intensive Care and Pain Medicine, Moenchebergstraße 41-43, D 34125 Kassel, Germany

**Keywords:** Euthanasia, Physician assisted dying, Palliative medicine, Willingness to hasten the patient’s death

## Abstract

**Background:**

Euthanasia and physician assisted suicide (PAS) are highly debated upon particularly in the light of medical advancement and an aging society. Little is known about the professionals’ willingness to perform these practices particularly among those engaged in the field of palliative care and pain management. Thus a study was performed among those professionals.

**Methods:**

An anonymous questionnaire was handed out to all participants of a palliative care congress and a pain symposium in 2013. The questionnaire consisted of 8 questions regarding end of life decisions. Proposed patient vignettes were used.

**Results:**

A total of 470 eligible questionnaires were returned, 198 by physicians, 272 by nurses. The response rate was 64 %. The majority of professionals were reluctant to perform euthanasia or PAS: 5.3 % of the respondents would be willing to perform euthanasia on a patient with a terminal illness if asked to do so. The reluctance grew in case of a patient with a non-terminal illness. The respondents were more willing to perform PAS than euthanasia. Nurses were more reluctant to take action as opposed to the physicians. The majority of the respondents would attempt to treat the patient’s symptoms first before considering life-ending measures. As regards any decision making process the majority would consult with a colleague.

**Conclusions:**

This is the first German study to ask about the willingness of professionals to take action as regards euthanasia and PAS without biased phrasing. As opposed to the general acceptance that is respectively high, the actual willingness to perform life-ending measures is low. The German debate on physician assisted suicide and its possible legalization should also incorporate clarifications regarding the responsibility who should eventually perform these acts.

## Background

Euthanasia and physician assisted suicide (PAS) are highly debated upon particularly in the light of medical advancement and an aging society [1]. In this paper the definition of euthanasia is: " A doctor intentionally killing a person by the administration of drugs, at that person’s voluntary and competent request" [2]. " PAS is a doctor intentionally helping a person to commit suicide by providing drugs for self-administration, at that person’s voluntary and competent request" [2]. So far Germany has not passed any legislation on euthanasia or PAS. However, legislative procedure is starting in autumn this year focusing on PAS. The German Society of Palliative Care, however, has taken a stand and declared their rejection of PAS [3, 4]. At the same time the support for euthanasia among the German public is growing [5].

But even in those countries that have legalized euthanasia or PAS there is an ongoing debate about the boundaries of these practices and questions regarding the responsibilities of physicians and nurses. Data from the Netherlands have shown that it is mostly general practitioners who perform euthanasia or PAS [6]. Those physicians that have admitted publicly to being involved in PAS in Germany were not palliative care specialist but practiced e.g. urology or psychiatry. Nevertheless it is the palliative care specialists, who are specifically trained in end of life care.

Various surveys regarding euthanasia and PAS have been performed [7–12]. However, only very little is known about the professionals’ willingness to perform these practices especially among those trained and involved in palliative care [9, 13–17]. Thus a study among professionals involved in the field of palliative care and pain management was performed. On the one hand the recruitment took place at a palliative care conference, which means only professionals with a current link to palliative care were included. Furthermore physicians were recruited at a pain symposium. In Germany, both pain medicine and palliative medicine are special qualifications. The special qualification pain medicine however, was introduced 5 years earlier. Both fields overlap and pain specialists have proven to play an essential role in the care of terminal patients. Symptom control at the end of life in Germany is a task that is now mainly performed by specialists rather than general practitioners as reported in countries such as the Netherlands [13, 18, 19]. In 2007 the “SAPV” (specialized out-patient palliative care) was introduced in the German code of social law thus aiming at establishing a comprehensive provision of palliative care for terminal patients [20]. Care is to be provided by multidisciplinary palliative care teams [21]. This particular group of professionals is also of special interest in the debate since some have declared euthanasia and PAS as possible outcomes of palliative care [22–24]. In the year before a study was performed at the same conference [25, 26]. However, that study focused on support of the legalization of euthanasia or PAS rather than exploring whether the respondents would actually be willing to perform the act.

## Methods

### Sample

The study is based on an anonymous questionnaire handed out to all participants of a palliative care conference and a pain symposium in 2013. Attendants were physicians and nurses. The concept of palliative care is an interdisciplinary one. Ethical decisions take place in a team approach thus acknowledging the role of the nurses in particular. Furthermore data from e.g. Belgium or the Netherlands has shown the central role of nurses in end of life practices such as assisted dying [27–30]. In 12 % of the cases it was the nurse who performed the life ending act [31]. This is a remarkable figure. Furthermore they play an essential role in the decision making process since they are often approached by both patients and physicians regarding end-of-life decisions [32, 33] This is why the study also included nurses.

### Instrument

The first part of the questionnaire regarded personal data asking about gender, age, occupation and special qualification in palliative care or pain medicine.

The questionnaire consisted of 8 questions on end-of-life (EOL) decisions: Concrete patient scenarios were used. They were adapted from a questionnaire by Seale [7], which was adapted from a large British study [34]. The British social attitudes survey has been used for many years and has established itself as a useful source of evidence [7]. The survey by Seale aimed at clarifying the stance of the British medical profession regarding the legalization of euthanasia or PAS. The main issue in our survey was whether the respondent is willing to perform euthanasia and physician assisted suicide (PAS) if asked to do so by their patient. Our questionnaire avoids the use of multiple hypothetical propositions, which has proven to make the results unreliable [35]. Knowledge of the definitions of euthanasia or PAS was not required. Instead the questionnaire explicitly named the actions to be taken by the physician. Thus avoiding the bias that comes with the use of words like “euthanasia”—from the Greek: “good death” or the German “Sterbehilfe”—“help to die”.

The first two questions focused on a patient with a terminal illness, questions 3 and 4 focused on a patient with a non-terminal illness. Additionally the illness was described as “painful” in order to hint at clear physical suffering [7]. The last 4 questions aimed at discovering details about the decision making process: consultation with a colleague, an attempt to treat the symptoms, duration of the treatment attempts (Table 1). Possible answers were “yes”, “no” and “I don’t know”.

**Table 1:** Questionnaire: willingness to perform euthanasia and PAS and details of the decision making process

1.	A patient has an incurable, painful illness, from which they will die, for example cancer. Would you fulfill his wish and end his life using medication?		
	Yes	No	I don’t know
2.	If this patient asks for it, would you give him lethal medication so that he can end his own life?		
	Yes	No	I don’t know
3.	A patient has an incurable, painful illness, from which they will not die. Would you fulfill his wish and end his life using medication?		
	Yes	No	I don’t know
4.	If this patient asks for it, would you give him lethal medication so that he can end his own life?		
	Yes	No	I don’t know
5.	I would make this decision by myself in order to not burden others		
	Yes	No	I don’t know
6.	I would not make a decision without consulting my colleagues		
	Yes	No	I don’t know
7.	I would first attempt to treat the patient’s symptoms on order to change his wish		
	Yes	No	I don’t know
8.	For this treatment attempt I would estimate the following amount of time		
	1 week	2 weeks	4 weeks

**Table 2:** Demographics of the respondents

		*n* (%)
Gender	Female	353 (75.1)
	Male	104 (22.1)
	Not specified	13 (2.8)
Age (years)	≤35	49 (10.4)
	36–45	97 (20.6)
	46–55	230 (48.9)
	56–65	83 (17.7)
	>65	9 (1.9)
	Not specified	2 (0.4)
Occupation	Nurses	272 (57.9)
	Physicians	198 (42.1)
Special qualification in palliative care	Yes	109 (55.1)
	No	89 (44.9)
Special qualification in pain medicine	Yes	60 (30.3)
	No	138 (69.7)
No special qualification in either palliative care or pain medicine	47 (23.7)	

### Data analysis

Statistical analysis was performed using SPSS Version 22.0 (IBM Corporation, Armonk, NY, USA). The significance level was set to p ≤0.05. Chi Square and exact Fisher tests were used to analyze bivariate relationships. For the dichotomous feature of a terminal or non-terminal illness the non-parametric McNemar test was used.

### Compliance with ethics guidelines

The study was approved by the ethics committee of the Ruhr University Bochum (Reg. no.: 4502/2012). Consent was given by the participants in completing the questionnaire.

## Results

A total of 470 eligible questionnaires were returned, 198 by physicians, 272 by nurses (Table 2). The response rate was 64 %.

### Euthanasia and PAS in case of a terminal illness

5.3 % of the respondents would be willing to perform euthanasia on a patient with a terminal illness if asked for it. 79.6 % would not be willing to do so and 14.7 % were undetermined.

The willingness to take action grew in case of PAS: 13 % of the respondents would be willing to perform PAS in a patient with a terminal illness. 66 % refused to do so, the proportion of respondents choosing “I don’t know” grew to 20.4 %.

### Euthanasia and PAS in case of a non-terminal illness

For a patient with a non-terminal illness the percentage of respondents willing to perform euthanasia decreased to 1.1 % (p ≤ 0,05).

The willingness to perform PAS in non-terminal illness was 3 %.

### Differences among physicians and nurses

Concerning a terminal illness physicians were significantly more prepared to take action in both euthanasia and PAS (p ≤ 0.05), see Table 3: In case of a terminal illness 7.1 % of the physicians were willing to perform euthanasia as opposed to 4 % of the nurses. At the same time 18 % of the nurses answered by “I don’t know”, while it was 11 % of the physicians. As regards PAS 15.7 % of the physicians were willing to take action vs. 11 % among the nurses. Again the nurses were more often undecided in their answer: 23.9 % chose “I don’t know”, vs. 15.7 % among the physicians.

**Table 3:** Responses of physicians and nurses questions 1 to 4

Question	Answer	Physicians *n* (%)	Nurses *n* (%)	Total *n* (%)
1. A patient has an incurable, painful illness, from which they will die, for example cancer. Would you fulfill his wish and end his life using medication?	Yes	14 (7.1)*	11 (4.0)	25 (5.3)
	No	164 (82.8)*	210 (77.2)	374 (79.6)
	I don’t know	20 (10.1)*	49 (18.0)	69 (14.7)
2. If this patient asks for it, would you give him lethal medication so that he can end his own life?	Yes	31 (15.7)*	30 (11.0)	61 (13)
	No	136 (68.7)*	174 (64.0)	310 (66)
	I don’t know	31 (15.7)*	65 (23.9)	96 (20.4)
3. A patient has an incurable, painful illness, from which they will not die. Would you fulfill his wish and end his life using medication?	Yes	4 (2.0)	1 (0.4)	5 (1.1)
	No	181 (91.4)	244 (89.7)	425 (90.4)
	I don’t know	23 (8.5)	23 (8.5)	36 (7.7)
4. If this patient asks for it, would you give him lethal medication so that he can end his own life?	Yes	8 (4.0)	6 (2.2)	14 (3)
	No	177 (89.4)	242 (89.0)	419 (89.1)
	I don’t know	13 (6.6)	22 (8.1)	35 (7.4)

*p ≤ 0.05

As regards a patient with a non-terminal illness again the physicians were more prepared to take action. However, the differences between physicians and nurses were not statistically significant.

### Differences among physicians with a special qualification in palliative care and pain medicine

Physicians with a special qualification in palliative care were more reluctant to hasten a patient’s death through euthanasia or PAS both in terminal and non-terminal illness (Table 4). Only 3.7 % of the physicians with special qualification in palliative care were willing to perform euthanasia in case of a terminal illness vs. 11.2 % of the physicians without this special qualification (p ≤ 0.001). For PAS in terminal illness, it was 11.9 % vs. 20.2 %.

**Table 4:** Responses of physicians with or without special qualification in palliative care or pain medicine

Question	Answer	Special qualification in palliative care *n* (%)	No special qualification in palliative care *n* (%)	Special qualification in pain medicine *n* (%)	No special qualification in pain medicine *n* (%)	No special qualification in either palliative care or pain medicine *n* (%)
1. A patient has an incurable, painful illness, from which they will die, for example cancer. Would you fulfill his wish and end his life using medication?	Yes	4 (3.7)**	10 (11.2)**	5 (8.3)	9 (6.5)	6 (12.8)
	No	99 (90.8)**	65 (73.0)**	49 (81.7)	115 (83.3)	35 (74.5)
	I don’t know	6 (5.5)**	14 (15.7)**	6 (10.0)	14 (10.1)	6 (12.8)
2. If this patient asks for it, would you give him lethal medication so that he can end his own life?	Yes	13 (11.9)	18 (20.2)	14 (23.3)	17 (12.3)	9 (19.1)
	No	76 (69.7)	60 (67.4)	39 (65.0)	97 (70.3)	32 (68.1)
	I don’t know	20 (18.3)	11 (12.4)	7 (11.7)	24 (17.4)	6 (12.8)
3. A patient has an incurable, painful illness, from which they will not die. Would you fulfill his wish and end his life using medication?	Yes	2 (1.8)	2 (2.2)	1 (1.7)	3 (2.2)	2 (4.3)
	No	103 (94.5)	78 (87.6)	56 (93.3)	125 (90.6)	39 (83.0)
	I don’t know	4 (3.7)	9 (10.1)	3 (5.0)	10 (7.2)	6 (12.8)
4. If this patient asks for it, would you give him lethal medication so that he can end his own life?	Yes	4 (3.7)	4 (4.5)	2 (3.3)	6 (4.3)	3 (6.4)
	No	99 (90.8)	78 (87.6)	54 (90.0)	123 (89.1)	40 (85.1)
	I don’t know	6 (5.5)	7 (7.9)	4 (6.7)	9 (6.5)	4 (8.5)

**p ≤0.001 (regarding physicians with and without special qualification in palliative care)

**Table 5:** Responses of physicians and nurses questions 5 to 8

Question	Answer	Physicians *n* (%)	Nurses *n* (%)	Total *n* (%)
5. I would make this decision by myself in order to not burden others	Yes	46 (23.2)	66 (24.3)	112 (23.8)
	No	133 (67.2)	170 (62.5)	303 (64.5)
	I don’t know	35 (7.4)	12 (6.1)	35 (7.4)
	Not specified	7 (3.5)	13 (4.8)	20 (4.3)
6. I would not make a decision without consulting my colleagues	Yes	123 (62.1)	157 (57.7)	280 (59.6)
	No	51 (25.8)	60 (22.1)	111 (23.6)
	I don’t know	31 (11.4)	15 (7.6)	46 (9,8)
	Not specified	9 (4.5)	24 (8.8)	33 (7.0)
7. I would first attempt to treat the patient’s symptoms on order to change his wish	Yes	147 (74.2)**	151 (55.5)	298 (63.4)
	No	24 (12.1)**	39 (14.3)	63 (13.4)
	I don’t know	21 (10.6)**	59 (21.7)	80 (17.0)
	Not specified	6 (3.0)**	23 (8.5)	29 (6.2)
8. For this treatment attempt I would estimate for the following amount of time	1 week	17 (11.5)**	35 (23.0)	52 (11.)
	2 weeks	33 (22.3)**	50 (32.9)	83 (17.7)
	4 weeks	87 (43.9)**	47 (30.9)	134 (28.5)
	Not specified	11 (7.4)**	20 (13.2)	31 (6.6)

**p ≤ 0.001

Physicians with a special qualification in pain medicine on the other hand were more prone to euthanasia or PAS in case of a terminal illness than physicians without this special qualification: 8.3 % were willing to perform euthanasia vs. 6.5 % among physicians without this qualification. In case of a non-terminal illness it was the other way around: physicians with special qualification in pain medicine were more reluctant to hasten a patient’s death through euthanasia or PAS.

Thirty-three physicians had the special qualification in both palliative care and pain medicine. Overall the differences between this group and the physicians that did not have both special qualifications were not statistically significant.

Physicians without any special qualification in either palliative care or pain medicine were more willing to perform euthanasia in both terminal and non-terminal illness. However, the differences were not statistically significant.

### Decision-making

The majority of the respondents would not make the decision about euthanasia or PAS by themselves (64.5 %) but would consult with a colleague first, 59.6 %. This was true both for physicians and nurses (Table 5).

Asked whether they would attempt to treat the symptoms before ending the patient’s life the majority answered “yes”: 63.4 %. The differences between physicians and nurses on this matter were significant: 74.2 % of the physicians chose to treat the patient, while it was 55.5 % of the nurses (p ≤ 0.001). 21.7 % of the nurses were undecided and chose “I don’t know”.

58.8 % of the physicians that would first attempt to treat the symptoms would estimate 4 weeks for the attempt to discover the conclusive patient’s wish. Among the nurses it was 30.9 % who would chose 4 weeks for the treatment attempt.

### Limitations

Given that the study focused on attendants of a palliative care and pain conference the reference group holds a certain bias. However, it was the aim of the survey to explore the attitudes of professionals engaged in the field of palliative care and pain management specifically. Because of this bias the results obtained do not reflect the general opinion of German physicians and nurses but provide an insight on the point of view of those professionals faced with end-of-life decisions in daily practice. No control group was surveyed, because we were only interested in the possible focus group.

Some of the practices the questionnaire asked about are currently illegal in Germany. This might prevent the respondents from giving honest answers. To reduce the reluctance to answer honestly the questionnaire was anonymous [36].

Additionally the questionnaire at hand avoids terminology that provides a certain bias itself (e.g. the term “euthanasia”). The patient scenarios used are adapted from a questionnaire by Seale and were translated to German [7]. Accordingly, our case vignettes referred to a painful terminal or non-terminal illness to focus on a physical symptom. Pain has proven to be a valid and quantifiable indicator for unbearable suffering in patients at the end of their life [37]. The patient scenarios used are limited: patients with other illnesses than cancer such as dementia (which has become more and more relevant in our aging society) were not included. Those scenarios are a complex field that should be investigated separately.

Regarding the statistical analysis a post hoc performed logistic regression model concerning age, occupation and special qualification proved no significant results. This was not unexpected regarding the sample and its structure. Nevertheless this might point to possible deficits in the strength of the statistical significance of the data at hand.

## Discussion

This is the first German study to ask about the willingness of professionals to take action as regards euthanasia and PAS without biased phrasing such as euthanasia from the Greek “help to die” in German “Sterbehilfe”. Only few studies focused on palliative care professionals in particular [38–40]. This adds to the current debate: numerous studies regarding the general acceptance of euthanasia and PAS have been conducted [7, 38, 41, 42]. A recent study among German physicians showed that already now without legalization of PAS or euthanasia both practices are performed by a minority [43]. This holds also true for members of the German Society for Palliative Care [38]. These findings are based on the EURELD-questionnaire, which focused on physicians’ and their colleagues’ experiences with patients that died within the last 12 months [33]. However, this questionnaire combines questions regarding euthanasia or PAS—which are currently illegal - with questions regarding established end of life care measures such as “withdrawing”, “withholding”, or “intensifying” treatment thus making answers dubious. Several studies have demonstrated that physicians have difficulties in discriminating between euthanasia and palliative sedation or euthanasia and intensified pain relief and lack knowledge regarding the procedure [19, 44–46]. Unlike the Eureld-questionnaire we used concrete patient vignettes to explore the willingness to act thus allowing a clear insight into the physicians’ willingness to actively end their patient’s life.

### General acceptance and willingness to act

The general support regarding the legalization of euthanasia and PAS is respectively high in Germany [12]. However, the willingness to perform these practices is low: only 5.3 % of the respondents would be willing to perform euthanasia on a patient with a terminal illness. This is in line with previous studies among professionals and medical students thus emphasizing that general acceptance and willingness to perform differ widely and have to be looked at independently [8, 11, 15, 29]. Similar results were reported from Finland: over time the attitude towards euthanasia became more positive, however, this change did not apply to the willingness to act [11]. This is also true in countries that have legalized euthanasia or PAS, such as Belgium: Even there the willingness to perform these practices is limited among general practitioners [13].

### Physicians vs. nurses

The differences in the attitudes of physicians and nurses are remarkable: Physicians are more willing to take action than nurses both in case of a terminal and a non-terminal illness. This result is surprising since former studies prompted to nurses being supportive of euthanasia and PAS [26, 29, 41, 42, 47]. However, a study among critical care nurses from Israel also found nurses to be supportive of PAS but only few agreed to participate in this act [48].

One reason for the nurses’ reluctance to take action might be that the nurses’ role in the performance of these end of life practices is still unclear even in countries that have legalized these practices [29]. However, in Germany a number of cases have been reported of nurses going beyond the professional and legal boundaries and administering drugs to end the patient’s life without the patient’s [49, 50]. These cases show the necessity to also investigate nurses regarding end of life questions.

In Belgium law requires the physicians to discuss the euthanasia request with the nursing team in charge (Chapter II section 3, § 2.4), which shows that nurses do play an important role in end of life practices. This is particularly true since it is often the nurses that patients talk to about their wishes [29]. The role of nurses in end of life decision-making is also mirrored in other studies. Nurses are involved both in the decision making process and the administration of the death hastening drugs [27].

In some cases euthanasia and PAS were even performed without request of the patient [27]. This does not seem to be a singular phenomenon: a Canadian study showed that about 63 % of the nurses asked would be willing to perform euthanasia without knowing the patient’s wish [16]. Among the physicians it was only one third that were willing to perform euthanasia without knowing the patient’s wish concerning an act of euthanasia [17]. A Belgian study based on a death certificate survey in 2007 identified 66 cases of euthanasia “without explicit request” by the patient [51, 52]. To further investigate what “without explicit request” meant another study was performed and those cases were analyzed once more [52]: The findings showed that most of these cases could not be described as “non-voluntary ending of life” thus the label “without explicit request” is misleading.

A study among 1509 Dutch nurses showed that the majority of hospital nurses did not accept inserting an infusion needle to administer a fatal dose as a task for nurses, serving on a review-board for euthanasia cases was accepted only by 45 % of the nurses asked [9]. A study from Spain found 51.5 % of the nurses willing to perform euthanasia if legalized [14]. However, about one third of those nurses could not identify a case of PAS correctly or report the legal status of PAS in Spain [14].

### Special qualification

In line with previous studies we found physicians with a special qualification in palliative care to be more reluctant to end their patient’s life [7, 53–55]. However, physicians with a special qualification only in pain medicine were more open to performing these acts.

Little is known about factors influencing a physician’s willingness to perform EOL practices. A Canadian study among physicians of various specialties showed that “perceived behavioral control” was one of the main determinants for the willingness to perform euthanasia [17]. Moreover former experience in the field of euthanasia and PAS has also proven to determine future willingness to take part in these practices [56].

### Differences in the willingness to perform euthanasia or PAS

Our results show more willingness to perform PAS than euthanasia among the professionals, thus leaving the final responsibility to the patient himself. 13 % of the respondents willing to perform PAS on a patient with a terminal disease is a remarkable amount. This is in line with the emphasis put on the patient autonomy [57]. However, previous studies in Spain and Northern Ireland found more willingness to be involved in euthanasia rather than PAS [14, 15]. This hints to a more paternalistic approach with euthanasia leaving it to the physician to take action and end the patients’ life rather than the patient ending his own life by taking the lethal medication.

These diverse interpretations of the performance of euthanasia and PAS can also be found in the Netherlands, where both practices are legal: PAS is perceived to leave more responsibility to the patient himself, safeguarding his autonomy while at the same time leaving less burden to the physician [57].

### Decision making process

One aspect that has not been in the focus of research yet is the final decision making process. Our results show that the majority of the health care professionals asked would interact with a colleague before granting a request to hasten the patient’s death: 59.6 % of the respondents would consult a colleague first before coming to a decision. The need for a second opinion is also demanded in countries that have legalized euthanasia or PAS, e. g. Belgium or the Netherlands, where the consultation of a second physicians or a euthanasia review committee is a procedural requirement [58].

Facing these legal regulations and European guidelines on palliative sedation, merely 60 % consulting others before life-ending decisions is a low figure [59]. However, this figure is in line with data from the Netherlands and Belgium indicating that the need for consultation is not accepted by all physicians [60–62]. In about 35 % of the cases of ending of life without explicit request by the patient no consultation took place in advance [6].

### Treatment attempt

Requests for euthanasia or PAS also occur in palliative care settings [63, 64]. Some argue that there is a “synergistic relationship” between palliative care and euthanasia or PAS, which means both of those practices could be embraced in the palliative care setting as possible therapeutic measures [22, 64]. The EAPC task force, however, argues clearly that euthanasia or PAS should not fall into the responsibility of palliative care [2]. The vital role of palliative care in the debate is also mirrored in our results with 63.4 % of the respondents wanting to attempt to treat the patient’s symptoms first before making any ultimate decisions. Some have even argued that in the absence of palliative care patients would not be able to make an informed meaningful decision [65, 66]. This however, has been disputed in the literature arguing that an autonomous decision regarding euthanasia or PAS is not limited to patients that are being treated in an ideal setting i.e. one that provides palliative care [67]. Nevertheless even liberal authors emphasize the importance of enhancing palliative care and its availability in the light of legalizing euthanasia or PAS [68].

A caveat is represented by studies demonstrating that the patients’ wish to die often changes over time and euthanasia requests are withdrawn [23, 63, 69]. A treatment attempt could buy some time to secure that the patient’s wish to die is not just temporary. Furthermore this also leaves room to provide the patients with more information regarding end of life, euthanasia or palliative sedation, which has proven to be important in their decision-making [70].

Regarding the length of the treatment attempt the majority of physicians estimated 4 weeks to be needed. Keeping in mind that depression can be a problem in patients at the end of their life [71] and that antidepressants take about 2 to 3 weeks to take effect this finding is not surprising.

## Conclusions

Our results demonstrate a profound problem in the actual discussion about PAS in Germany: if PAS were allowed, who should perform it. General practitioners are not trained to perform any form of life-ending procedures. This has been documented extensively by studies from Belgium and the Netherlands, where false drugs were selected, euthanasia was mistaken for palliative sedation and vice versa [44, 61, 72–75]. On the other hand, palliative care physicians are reluctant to perform euthanasia or PAS. So far public debate has disregarded the central question: who should train doctors to perform PAS correctly.

Thereby our study should stimulate an ongoing discussion before opening political discussions to allow or prohibit PAS for physicians in Germany.

## Abbreviations

PAS: Physician assisted suicide
EOL: End of life
